# Management of Thoracic Complications After Supracostal Mini-Percutaneous Nephrolithotomy in Pediatric Patients: An Initial Experience

**DOI:** 10.7759/cureus.99058

**Published:** 2025-12-12

**Authors:** Qudrat Ullah, Sajid Malik

**Affiliations:** 1 Urology, Institute of Kidney Diseases, Peshawar, PAK

**Keywords:** chest tubes, hemothorax, hydrothorax, percutaneous nephrolithotomy, thoracentesis

## Abstract

Objective

The main objective of this study is to assess the frequency and management of thoracic complications following supracostal mini-percutaneous nephrolithotomy (mini-PCNL) in pediatric patients.

Methods

This retrospective cross-sectional study was conducted in the Department of Urology, Institute of Kidney Diseases, Peshawar, Pakistan, from June 2017 to December 2019. A total of 80 pediatric patients (52 males, 65%, and 28 females, 35%) who underwent supracostal mini-PCNL were included. Patients were categorized according to the level of puncture: Group 1 (between the 11th and 12th ribs; n = 62, 77.5%), Group 2 (between the 10th and 11th ribs; n = 15, 18.75%), and Group 3 (between the 9th and 10th ribs; n = 3, 3.75%). Postoperative thoracic complications, including hydrothorax and hemothorax, were documented and managed either conservatively, by needle aspiration, or by intercostal chest tube insertion.

Results

Among the 80 patients, 12 (15%) developed hydrothorax. Although thoracic complications were observed in 6/62 (9.6%) in Group 1, 3/15 (20%) in Group 2, and 3/3 (100%) in Group 3, the interpretation of the 100% complication rate in Group 3 should be made with caution, because this group contained only three patients, limiting the statistical power despite the anatomical plausibility of higher complications at higher intercostal levels. Of the affected patients, six (50%) were managed conservatively, four (33.3%) required needle aspiration, and two (16.6%) underwent intercostal chest tube insertion. The mean hospital stay among patients with thoracic complications was 2.3 days.

Conclusion

The likelihood of thoracic complications following supracostal mini-PCNL in pediatric patients increases with higher intercostal access. While punctures above the 10th rib carry a 100% risk of hydrothorax, most cases can be managed conservatively through timely recognition and multidisciplinary collaboration among urologists, anesthetists, and pulmonologists. A meticulous surgical approach and careful perioperative monitoring significantly reduce morbidity.

## Introduction

Urolithiasis is a common condition worldwide, with an increasing prevalence in the pediatric population. In Asia, its incidence is rising, partly due to sedentary lifestyles, dietary changes, and westernized eating habits [1]. Kidney stones can significantly affect the quality of life, causing pain, urinary tract obstruction, and recurrent infections, and they impose a substantial economic burden on healthcare systems [2].

Percutaneous nephrolithotomy (PCNL) has become the preferred treatment for large or complex renal stones, including in pediatric patients, due to its high stone clearance rates and minimally invasive nature. However, PCNL, particularly via the supracostal approach, carries potential complications, such as hydrothorax, hemothorax, and injury to surrounding organs, which may be more pronounced in children due to anatomical considerations. Understanding the prevalence and management of these thoracic complications is essential for safe and effective pediatric stone management [2].

The management of kidney stones depends on their size, type, and location [3]. Stones may be classified as calcium oxalate, uric acid, struvite, cystine, or mixed types, each with distinct pathophysiological characteristics and clinical implications. Treatment options range from conservative medical therapies and extracorporeal shock wave lithotripsy (ESWL) to endourological procedures, such as ureterorenoscopy (URS), retrograde intrarenal surgery (RIRS), and PCNL, as well as open or laparoscopic surgery for complex cases. Among these, PCNL has become the gold standard for managing large, complex, or recurrent stones due to its high stone clearance rates and minimally invasive nature. However, PCNL, especially via the supracostal approach, carries a risk of thoracic complications, including hydrothorax, hemothorax, and pneumothorax, which may occur when the pleura is breached during access, particularly in pediatric patients [4].

There are several treatment options for pediatric renal stones, including ESWL, flexible ureterorenoscopy (f-URS), and PCNL. Treatment selection depends on stone size, location, composition, and the child’s anatomical and clinical status. ESWL is less effective for large or hard stones, while f-URS may be limited by small ureteral caliber in children, reduced maneuverability, and the risk of ureteral trauma. Mini-PCNL has emerged as a preferred option for children with larger stone burdens because it offers higher stone-free rates, allows direct access to all calyces, and minimizes renal trauma through the use of smaller tract sizes. Supracostal access is one approach used to reach upper-pole or complex stones; however, it may carry a higher risk of thoracic complications compared with subcostal access. Because data on thoracic complications in pediatric supracostal mini-PCNL remain limited, this study aims to evaluate their frequency and management in our pediatric population [5].

Supracostal access during PCNL provides a direct route to the upper pole and facilitates treatment of complex stones; however, it is associated with a recognized risk of thoracic complications, most notably hydrothorax, due to the anatomical proximity of the pleura to the upper pole of the kidney [6]. The likelihood of pleural violation increases with higher intercostal puncture sites, as demonstrated in previous anatomical and clinical studies [7]. These risks emphasize the importance of careful access selection, awareness of pleural anatomy, and meticulous technique, particularly in pediatric patients, who have less respiratory reserve [8].

The goal of this retrospective study is to evaluate the frequency and management of thoracic complications, primarily hydrothorax and hemothorax, associated with supracostal mini-PCNL in pediatric patients. These complications arise mainly from inadvertent pleural violation due to the close anatomical relationship between the pleura and the upper pole of the kidney. Prior studies have reported that the risk of pleural complications increases with higher intercostal access during PCNL, particularly when access is obtained above the 11th rib [6]. Because children have smaller thoracic dimensions and reduced pulmonary reserve, thoracic complications may pose a greater risk in this population. Therefore, understanding the incidence and clinical management of these complications is essential to optimizing outcomes in pediatric supracostal mini-PCNL.

Surgical anatomy

In pediatric patients, the lungs and pleura occupy a smaller thoracic cavity, and their position relative to the ribs differs from that in adults. The lungs are enclosed in a potential space lined by visceral and parietal pleura. In children, the lower border of the parietal pleura reaches approximately the medial half of the 12th rib along the mid-scapular line, with an oblique course from the eighth rib laterally to the 12th rib, and extending from the midclavicular line to the midaxillary line around the 11th rib [9]. Awareness of these pediatric-specific anatomical landmarks is essential to minimize thoracic complications, such as hydrothorax, during supracostal mini-PCNL.

Objectives

The main objective of this study is to assess the frequency and management of thoracic complications following supracostal mini-PCNL in pediatric patients.

## Materials and methods

This retrospective cross-sectional study was conducted in the Department of Urology, Institute of Kidney Diseases, Peshawar, Pakistan, between June 2017 and December 2019. Ethical approval was obtained from the institutional review board. Since this was a review of existing patient records, individual informed consent was not required, according to institutional policy. A total of 80 pediatric patients who underwent single-tract supracostal mini-PCNL during the study period were included.

The inclusion criteria were patients younger than 16 years with staghorn stones, a stone burden larger than 2 cm, or those who had failed ESWL. Exclusion criteria included patients unfit for anesthesia, those with coagulation disorders, active pulmonary disease, or cases requiring multiple-tract PCNL. These patients were excluded to avoid confounding factors that could increase the risk of thoracic complications, ensuring that the analysis focused specifically on complications associated with single-tract supracostal access.

After applying the inclusion and exclusion criteria, all eligible patients underwent mini-PCNL via supracostal access, performed by a single experienced surgeon. Supracostal access was indicated in patients with upper-pole, complex, or large renal stones, where subcostal access was insufficient. Preoperative computed tomography of the kidneys, ureters, and bladder (CT KUB) was used to evaluate stone location, renal anatomy, and the safety of the proposed access tract. Although supracostal access above the 11th rib was commonly required, the exact puncture site was individualized based on imaging and intraoperative fluoroscopic guidance; ultrasound was not used due to center protocols and surgeon experience, but may be considered in future studies to further reduce thoracic risks.

Following induction of general anesthesia, a ureteral catheter was inserted, and the patient was placed in the lithotomy position, then repositioned prone. The selected calyx was accessed under fluoroscopy after retrograde contrast opacification. Tract dilatation was performed up to 16 Fr, allowing placement of a 16 Fr cannula and introduction of a 12 Fr nephroscope. Stone fragmentation was performed using the Swiss LithoClast® (EMS Electro Medical Systems, Nyon, Switzerland) pneumatic lithotripter at standard pediatric settings. The pelvicalyceal system (PCS) was carefully inspected for residual stones or injury, and fluoroscopy confirmed stone clearance. Finally, an antegrade double-J (DJS) stent was inserted over a glidewire for postoperative drainage.

Patients were categorized into three groups based on the supracostal puncture level selected during surgery (between the 11th-12th ribs, 10th-11th ribs, or 9th-10th ribs). The access site was chosen based on preoperative CT KUB, considering stone location, calyceal anatomy, kidney orientation, and safety of the puncture tract, with final confirmation under fluoroscopy. Postoperatively, chest X-rays were performed in all patients to detect thoracic complications, such as hydrothorax, and additional ultrasound or CT imaging was obtained only if clinical signs or symptoms were present. Management was stepwise: patients were initially treated conservatively with head-end elevation, analgesics, antibiotics, restricted intravenous fluids, diuretics, and close monitoring of vital signs. If symptoms worsened, needle aspiration using an 18-G needle at the second intercostal space in the midclavicular line was performed. Patients who did not improve or had significant collections underwent intercostal chest tube insertion in the triangle of safety.

Stone clearance was confirmed intraoperatively through direct nephroscopic visualization and fluoroscopy to detect any residual fragments.

## Results

A total of 80 pediatric patients underwent supracostal mini-PCNL during the study period. The mean age of the patients was 8.6 ± 2.4 years (range: 6 months-14 years). There was a male predominance, with 52 (65%) males and 28 (35%) females. The right kidney was affected in 48 (60%) cases, and the left in 32 (40%). The mean stone size was 2.6 ± 1.2 cm. Regarding stone location, pelvic stones were the most common (41.2%), followed by upper calyceal stones (21.2%), lower calyceal stones (15%), staghorn stones (12.5%), and proximal ureteric stones (10%) (Table 1).

**Table 1:** Demographics and clinical characteristics of the patient sample (n = 80) Baseline demographic data, side of involvement, stone location, and distribution of patients among supracostal access groups undergoing mini-percutaneous nephrolithotomy (mini-PCNL).

Variables	Frequency (n = 80) (%)
Age	6 months to 14 years
Gender (n, %)	
Male	52 (65%)
Female	28 (35%)
Laterality (n, %)	
Right	48 (60%)
Left	32 (40%)
Stone Location (n, %)	Pelvic stone - 33 (41.2%)
	Upper calyx - 17 (21.2%)
	Lower calyx - 12 (15%)
	Staghorn stones - 10 (12.5%)
	Proximal ureteric stone - 8 (10%)
No. of patients in different groups (n, %)	
Group 1	62 (77.5%)
Group 2	15 (18.5%)
Group 3	3 (3.75%)

Patients were categorized into three groups based on the level of supracostal puncture: Group 1 (11th-12th ribs, n = 62), Group 2 (10th-11th ribs, n = 15), and Group 3 (9th-10th ribs, n = 3).

Thoracic complications were observed in 12 patients (15%), which is consistent with previously reported rates for supracostal access in pediatric PCNL, where the proximity of the pleura increases the risk of hydrothorax. The higher complication rate in Group 3 (puncture above the 10th rib) reflects the anatomical risk associated with very high intercostal access, rather than the procedure itself, and these cases represented only three children with complex upper-pole stones requiring higher access. Importantly, most complications were mild hydrothorax and were managed conservatively, without long-term morbidity (Table 2).

**Table 2:** Various groups and chest complications Frequency of thoracic complications according to intercostal puncture level, showing an increased risk with higher supracostal access.

S. No.	Groups: Site of puncture	Frequency of complications in each group (%)
1	Group 1: between 11th and 12th rib	6 (9.6%)
2	Group 2: puncture between 10th and 11th rib	3 (20%)
3	Group 3: between 9th and 10th	3 (100%)

Of the 12 patients who developed thoracic complications, half (n = 6, 50%) were managed conservatively, four patients (33.3%) required needle aspiration for symptomatic hydrothorax, and two patients (16.6%) underwent intercostal chest tube insertion for hemothorax. The mean hospital stay for patients requiring management of thoracic complications was 2.3 days (Table 3).

**Table 3:** Statistics of treatment modalities and hospital stay Management approaches for thoracic complications, including conservative care, needle aspiration, and intercostal chest intubation, with the corresponding mean hospital stay.

Mode of treatment	Reason for treatment	Frequency of patients treated (%)	Stay in hospital (mean, days)
Conservative	Asymptomatic hydrothorax	6 (50%)	2
Needle aspiration	Symptomatic hydrothorax	4 (33.3%)	2
Intercostal chest intubation	Hemothorax	2 (16.6%)	3

## Discussion

A variety of renal and ureteral disorders that cannot be adequately treated by other methods require percutaneous renal surgery. The ureteropelvic junction (UPJ), the proximal ureter, and upper-pole calculi can all be directly accessed via the supracostal technique. By positioning the nephroscope along the longitudinal axis of the kidney, the surgeon can minimize bleeding by lowering the torque that the rigid nephroscope exerts. After preoperative assessment and preparation, the patient is placed in the prone position for the procedure with a ureteric catheter in the proximal ureter, taking all necessary precautions to avoid injury to the spine and limbs. The PCS is visualized with urografin diluted in 10 mL of normal saline; after opacification, the site of puncture is decided with preoperative assessment of kidney anatomy in mind [10]. The author uses a 16-G spinal needle for puncture once the glidewire is stabilized in the PCS or ureter, then single-step dilatation is performed, and a metallic Amplatz 17 Fr sheath is placed in the PCS. Stone fragmentation is performed using a pneumatic LithoClast, and stone retrieval is done with wash and bifrong forceps [11].

During the procedure, in close collaboration with an anesthetist, oxygen saturation and positive airway pressure are monitored, and sometimes ultrasound and fluoroscopy are used to assess the level of fluid in the pleural space [12]. Blood appears more hyperechoic than water on an ultrasound scan [13]. Fourteen patients who developed hydrothorax or hemothorax postoperatively were treated either conservatively, by needle aspiration, or by intercostal chest intubation [14-16]. These treatment options depend on the patient's clinical status, including oxygen saturation, air entry, and the volume and type of fluid in the pleural space [17].

Six patients with hydrothorax were managed conservatively in the urology ward, where they were monitored with daily chest X-rays to assess resolution. Their clinical status, including respiratory rate, chest expansion, air entry, and oxygen saturation, was closely observed. If the patient’s condition deteriorated or the hydrothorax failed to improve, they were then treated with either needle aspiration or intercostal chest intubation [18,19]. Four patients with clear fluid in the pleural space who were symptomatic, or whose hydrothorax did not resolve conservatively, required needle aspiration on postoperative days 1-2. Additionally, two patients who developed hemothorax underwent intercostal chest intubation on postoperative day 1 [20,21].

In our study, the overall incidence of thoracic complications was 15%, and all cases occurred in patients who underwent puncture above the 11th rib, with the highest rates observed in those requiring very high intercostal access. This pattern closely aligns with the findings of Shaban et al., who reported that supracostal punctures above the 11th rib carry a significantly higher risk of hydrothorax and pleural injury, including cases requiring intercostal tube placement. Similarly, Shaban et al. demonstrated that procedures involving nephrostomy tubes are associated with an increased likelihood of hydrothorax compared with tubeless techniques. These published observations further support our results, reinforcing that the level of supracostal puncture and the presence of nephrostomy drainage are major determinants of pleural complications [22]. In a prospective study by Sukumar et al. involving 110 supracostal PCNL procedures in the 11th intercostal space, 101 patients had only one access, while nine patients required additional access to the kidney for stone clearance [23]. Overall, stone clearance was 86.4% with PCNL alone, increasing to 97.3% with subsequent procedures. Two patients experienced intraoperative hemorrhage, one experienced perinephric fluid collection, 10 experienced thoracic problems, and two experienced sepsis. All patients with complications were managed conservatively, with hospital stays ranging from 2 to 15 days.

Our study shows that supracostal puncture between the 11th and 12th rib for mini-PCNL is a safe procedure, with a risk of chest complications of 9.6% if surgical boundaries are not violated. The thoracic complications associated with supracostal PCNL can be managed in the urology ward, with consultation from a pulmonologist. As the study has a small sample size, it requires further randomization to fully understand the management of thoracic problems and the safety of supracostal mini-PCNL.

The management of chest complications depends on the patient’s symptoms, the nature of the pleural fluid, and the response to conservative therapy. Although supracostal mini-PCNL remains an effective option for managing large or complex stones in children, it is important to acknowledge alternative modalities. Supine PCNL has been associated with comparable stone-free rates and may offer easier airway access for anesthesia, but studies show that thoracic complications, while uncommon, can still occur, particularly with upper-pole access. f-URS with laser lithotripsy is a less invasive alternative, but its utility in children is limited by smaller ureteral calibers, reduced maneuverability, and lower efficacy for larger stone burdens; however, it carries virtually no risk of thoracic complications. Similarly, ESWL avoids pleural injury altogether, but is less effective for stones larger than 2 cm or with complex anatomy. Considering these limitations, supracostal mini-PCNL remains a justified choice in appropriately selected pediatric patients, provided that it is performed with meticulous technique and multidisciplinary collaboration among urologists, anesthetists, radiologists, and pulmonologists.

## Conclusions

Supracostal mini-PCNL provides a direct approach for accessing upper-pole or complex stones in pediatric patients. Our study demonstrates that, while the procedure is effective for stone clearance, it carries a measurable risk of thoracic complications, particularly with higher intercostal access above the 10th rib. Most complications in our cohort were mild and managed successfully with conservative measures or minor interventions, such as needle aspiration or intercostal chest tube insertion. The safest access was between the 11th and 12th ribs, associated with minimal morbidity and a short hospital stay.

These findings highlight the importance of careful patient selection, individualized puncture planning, and multidisciplinary perioperative support to mitigate risk. Although not a randomized trial, this retrospective review provides observational evidence that, with adherence to anatomical landmarks, meticulous surgical technique, and vigilant intraoperative monitoring, supracostal mini-PCNL can be performed safely and effectively in selected pediatric patients. Alternative modalities, including f-URS or supine PCNL, may be considered in centers with the necessary expertise to further reduce pleural risk.
